# Anemia and Iron Deficiency in Adolescent School Girls in Kavar Urban Area, Southern Iran

**Published:** 2011-02-01

**Authors:** M Ramzi, S Haghpanah, L Malekmakan, N Cohan, A Baseri, A Alamdari, N Zare

**Affiliations:** 1 Hematology Research Center, Shiraz University of Medical Sciences, Shiraz, Iran; 2 Nephrourology Research Center, Shiraz University of Medical Sciences, Shiraz, Iran; 3 Statistical Department, Shiraz Medical School, Shiraz University of Medical Sciences, Shiraz, Iran

**Keywords:** Adolescent, Anemia, Iron deficiency, Iran

## Abstract

**Background:**

Anemia is one of the most common public health problems especially in developing countries. We investigated the prevalence of anemia, iron deficiency anemia and related risk factors in adolescent school girls in Kavar urban area in southern Iran.

**Methods:**

A total of 363 adolescent school girls were evaluated by a cross sectional study. Socioeconomic, demographic and related risk factors were obtained by a questionnaire. Hematological parameters and serum iron indices were measured.

**Results:**

There were 21 cases of anemia (5.8%), 31 (8.5%) iron deficiency and 6 (1.7%) iron deficiency anemia.  Most of anemic girls (85.7%) had mild anemia.  MCV, TIBC, age, and BMI had statistically significant relationship with hemoglobin. Only parasites infestation in the last three months had a 6.83 times more risk of anemia than those without this history (95% CI, 1.66-28.11).

**Conclusion:**

The prevalence of anemia and iron deficiency anemia in this study were substantially less than what reported in many other regions of Iran as well as other developing countries. It seems that related implemented strategies in the recent years have been successful. More especial attention to prevention of parasite infestation should be considered in this area.

## Introduction

Anemia is one of the most common public health problem worldwide and especially in developing countries. Based on the World Health Organization (WHO) criteria, more than two billion people globally and 149 million people in the Eastern Mediterranean Region (EMRO) are estimated to be anemic. The most common type of nutritional anemia is iron deficiency anemia which is approximately responsible for 50% of all anemia.[1][2][3][4][5] The major consequences of anemia are increased risk of maternal and child mortality followed by negative effects on physical and mental development of children and decreased learning and work capacity and influencing on reproductive health in adolescents and adults.[6][7] Anemia has been a major public health concern in children and pregnant women especially in developing countries. There are many studies with regard to these high risk groups. Adolescents make up roughly 20% of world population and even higher proportion in developing countries. It seems that adolescent girls are also at increased risk of anemia due to period of rapid growth and developmental process of adolescence which cause higher requirement on both micro and macronutrients especially in girls who attend menarche.[8][9] In addition, iron status and hemoglobin concentration in this group could be a predisposing factor for maternal anemia.[3][10]

Due to different socioeconomic, economical and other influencing factors, the epidemiology of anemia varies among different regions. This study was undertaken to determine the prevalence of iron deficiency and anemia as well as related risk factors among adolescent urban school girls in Kavar, Fars Province, southern Iran.

## Materials and Methods

Considering P=27%, d=5.6%, CI=95% and design effect=1.5, 363 adolescent school girls aged 10-19 years were evaluated by a cross sectional study in November-December 2008 in Kavar urban area located in Fars Province in southern Iran.The term adolescent defined by WHO includes persons aged between 10 to 19 years old.[11] Participants were selected by two stage random sampling design; four middle and four high schools selected by cluster random sampling as the first-stage unit. At the second stage, school girls were selected by stratified random sampling in each unit. Ethics Committee of Shiraz University of Medical Sciences approved the study protocol. Written informed consents were obtained from all participants as well as parents-teachers association of each school. Interviews were carried out by expert personnel. Data were collected by a designed data gathering form. Investigated variables included age, father and mother educational level, family size, age at menarche, history of excessive menstrual bleeding, vegetarian or not vegetarian diet, drinking tea 30- minutes before or after eating food, history of parasitic infestation in the last three months and use of iron supplement. Height and weight of the students were measured and Body Mass Index (BMI) was calculated as weight in kilograms divided by square of height in meters. BMI was subdivided into low (<18.5 kg/m(2)), normal (18.5-24.9 kg/m(2)) and high (≥ 25 kg/m2) according to WHO criteria.[12] All students were referred to a medical laboratory in Kavar for blood sampling. Five ml venous blood was taken from each student to measure hemoglobin and other hematological parameters using a hematology analyzer (Mindray BC-3000 plus, China) as well as iron indices including, serum ferritin (SF), serum iron and total iron binding capacity (TIBC). SF was measured by radioimmunoassay method (Beckman-Coulter Immunotech Kit, Czech). Serum iron and TIBC were determined by a colorimetric procedure (Pars Azmoon Kit, Iran). Anemia was considered with cut off point for hemoglobin level < 12 g/dL,[13] the severity of anemia was categorized as mild (10-12 g/dL), moderate (7-10 g/dL) and severe (<7 g/dL).[14] Iron deficiency was determined as serum ferritin concentration less than 12 μg/L; and iron deficiency anemia was defined as anemia with serum ferritin concentration less than 12 μg/L.

Correlations between hemoglobin and serum ferritin and TIBC were evaluated by Pearson's correlation test. Independent relationship of hemoglobin concentration with MCV, serum ferritin, BMI, age, and TIBC was assessed by stepwise multiple regression analysis. Logistic regression was performed to determine the association of anemia with various factors including school grade, parents' educational level, family size, status of menarche, vegetarian diet, drinking tea in a 30-minutes period of eating food, history of parasite infestation in the last three months and use of iron supplement. P-value < 0.05 was considered statistically significant. Statistical analysis was done with SPSS software (v. 15, SPSS Inc, Chicago, IL, USA).

## Results

Mean age of the participants was 14.63±1.72 years. 52.2 % (188) of girls were in late adolescence (15-19 years). Majority of girls were in families with parents' educational level of incomplete secondary level (65.5% of fathers and 71.7% of mothers). 82.6% (300) of girls attained menarche. Seven subjects (1.9%) were vegetarian. 19.8% (72) had habit of drinking tea in a 30-minutes period of eating food. History of parasite infestation in the recent three months was positive in 3% (11) of them. Iron supplementation was used in 46.3% (168) of participants.

Hematological and iron indices are summarized in Table 1. Twenty one girls (5.8%) had anemia (Hb<12), 31 subjects (8.5%) iron deficiency (serum ferritin< 12) and 6 cases (1.7%) iron deficiency anemia (Hb<12 and serum ferritin< 12). Most of the anemic girls (18 girls, 85.7%) were in the mild range of anemia, only three of them had moderate anemia and severe anemia was not seen. There was not significant correlation between hemoglobin concentration and serum ferritin. (r=-0.093, p=0.078), but a significant negative correlation between hemoglobin concentration and TIBC (r=-0.397, p<0.001).

As presented in Table 2; MCV, TIBC, age, and BMI had statistically significant relationship with hemoglobin. The associations of socioeconomic and demographic factors with anemia (hemoglobin concentration) evaluated by univariate logistic regression analysis were shown in Table 3. From all factors,only parasite infestation in the last three months was found to contribute with a 6.83 times more risk of anemia than those without a history of parasite infestation in the last three months (95% CI, 1.66-28.11).

**Table 1 :** Hematological and iron indices in adolescent girls in Kavar, southern Iran

**Variables**	**Minimum**	**Maximum**	**Mean**	**Standard deviation**
Hb (g/dl)	9	20	13.89	1.19
HCT (%)	28.20	62.30	40.38	3.01
MCV (fl)	56.50	100.8	83.87	8.88
MCH (pg)	18.60	36.10	28.85	3.58
MCHC (g/dl)	31	37.50	34.33	1.12
RBC/µ	3.72^a^10^6^	7.66^a^10^6^	4.85^a^10^6^	0.5^a^10^6^
Serum iron (µg/dl)	25	171	88.88	23.53
Serum ferritin (ng/ml)	1	285.4	41.77	32.34
TIBC (µg/dl)	321	518	360.71	37.03

^a^ Hb: hemoglobin, HCT: hematocrit, MCV: mean corpuscular volume, MCH: mean corpuscular hemoglobin, MCHC: mean corpuscular hemoglobin concentrate, RBC: red blood cells count, TIBC: total iron binding capacity.

**Table 2 :** Stepwise multiple regression for hemoglobin concentration of adolescent girls in Kavar, southern Iran

**Variables**	**B**	**β**	**T**	**P-value**
MCV (fl)^a^	0.074	0.557	12.98	<0.001
TIBC (µg/dl)^b^	-0.006	-0.187	-4.369	<0.001
Age (years)	-0.071	-0.105	-2.526	0.012
BMI^c^	0.024	0.084	2.012	0.045

^a^ Multiple r, 0.652; R(2), 0.425; adjusted R(2), 0.419; F-ratio, 65.287; (df=4); p<0.001MCV: mean corpuscular volume

^b^ TIBC: total iron binding capacity

^c^ BMI: body mass index

**Table 3 :** Univariate logistic regression analysis for risk factors of anemia in adolescent girls in Kavar, southern Iran.

**Variables**	**Total N=363**	**OR**	**CI for OR lower-upper**	**P-value**
Father education:				
Graduate and above	10	Reference	-	-
Secondary school	56	0.576	0.258-3.06	0.999
Primary and middle school	235	1.02	0.823-5.07	0.917
Illiterate	58	1.58	0.714-2.05	0.876
Mother education:				
Secondary school	19	Reference	-	-
Primary and middle school	256	0.919	0.179-4.72	0.919
Illiterate	82	0.382	0.078-1.862	0.234
School grade:				
Middle	157	Reference	-	-
High	205	0.681	0.282-1.646	0.393
Family size:				
1-5 children	264	Reference	-	-
16-11 children	93	2.25	0.91-5.52	0.07
Status of menarche:				
No	56	Reference	-	-
Yes	300	0.781	0.253-2.414	0.668
Menstruation duration:				
<7 days	149	Reference	-	-
7-15 days	155	1.78	0.64-4.95	0.267
Diet:				
Non vegetarian	321	Reference	-	-
Vegetarian	7	0.9	0.11-7.13	0.921
Habit of drinking tea:				
No	274	Reference	-	-
Yes	72	1.2	0.42-3.4	0.727
Parasite infestation:				
No	327	Reference	-	-
Yes	11	6.83	1.66-28.11	0.008
Iron supplement:				
No	181	Reference	-	-
Yes	168	1.47	0.603-3.583	0.397

## Discussion

In our study, the prevalence of anemia, iron deficiency, and iron deficiency anemia among adolescent school girls were 5.8%, 8.5%, and 1.7% respectively. Based on previous studies in Northern and Western Iran, the prevalence of anemia, iron deficiency, and iron deficiency anemia in the similar age groups were reported as 7.5%-21.4%, 23.7%-44%, and 2.5%-13.6% respectively.[15][16] In our region in southern Iran, the prevalence of iron deficiency anemia in Turkish nomads was 17.7%.[17] This figure in Lor nomads in southern Iran was 17.7% too.[18] A prevalence of 59.8% was reported for anemia in adolescent girls of rural Wardha,[19] 15.8% in adolescent rural Amazonians,[20] 27% among adolescent schoolgirls in periurban Bangladesh,[21] and 29% in adolescent girls in the urban slums of Vellore, south India.[22] Leenstra et al.[23] reported a prevalence of 21.1% for anemia and 19.8% for iron deficiency in adolescent schoolgirls in Western Kenya. WHO reported a prevalence of 27% for anemia in developing countries and 6% in industr lized ones.[9] The prevalence of anemia and iron deficiency in this study is lower than what reported previously from this age group in other regions in Iran as well as the reports of other developing countries. This result is hopeful and possibly is related to the recent efforts of the Ministry of Health by implementation of many related projects including the increase of absorption and bioavailability of iron such as fortification of bread with iron, using iron supplement in adolescent girls and promoting the knowledge of population regarding how to increase the absorption and bioavailability of iron. However, the reasons as probable causes of this low prevalence may be as follows: First, we just studied the urban population in Kavar town with a better health condition in comparison to rural population. Second, our study was limited to the adolescent girls in schools which they have usually higher socioeconomic status and knowledge than those not attending the schools.

Only 29% of all anemic subjects were iron deficiency anemia. Also we did not find any significant correlation between hemoglobin concentration and serum ferritin suggesting that iron status was not likely an important determinant factor of anemia in the studied population. These results are in contrast to what reported by Hashismue et al.[24] who found asignificant positive correlation between hemoglobin concentration and serum ferritin among school children in the Aral Sea Region of Kasakhestan (r=0.275, p= 0.001). Also it differs from the results of the report by Karimi et al.[25] who showed a significant positive correlation between hemoglobin concentration and serum ferritin in pregnant women in Southern Iran (r= 0.76, p=0.01). Maybe children and pregnant women are more prone to iron deficiency than adolescent girls and these discrepancies could be interpreted by different studied populations.

Factors influencing hemoglobin concentration were MCV, TIBC, age, and BMI. Hashismue et al.[24] documented a similar significant relation of hemoglobin with MCV and age but it deferred regarding TIBC and BMI. A higher prevalence of anemia was also detected in relationship with lower BMI in the adults group ranged 16-70 years in North India[8] however, age and BMI did not contribute significantly with anemia in rural adolescent girls in Wardha.[19] Also in non-pregnant women in rural population in Bangladesh, the prevalence of anemia was not associated with age.[3]

Considering socioeconomic, demographic and other risk factors, the association of anemia with various factors including educational level of girls, parents' educational level, family size, status of menarche, having a vegetarian diet, drinking tea in a 30-minutes period of eating food, history of parasite infestation in the last three months and use of iron supplement were evaluated. From all investigated factors, only parasite infestation in the last three months was associated with anemia. This relationship has been shown also by Kaur et al.[19] in rural adolescent girls of Wardha. Leenstra et al.[23] showed that malaria and shistosomiasis were the main risk factors for anemia in young adolescent girls too. Regarding socioeconomic status, in contrast to our results, it was associated with a higher prevalence of anemia in many other studies.[2][3][8] Considering menstruation status, in contrary to our findings, a higher prevalence of anemia was reported in the adolescent girls who had attained menarche.[19][23]

Fortunately the prevalence of anemia, iron deficiency, and iron deficiency anemia in adolescent girls of Kavar urban area were substantially less than what reported from many other regions of Iran as well as other developing countries. Iron deficiency is not the major underlying cause of anemia in this area. It seems that related implemented strategies including increasing absorption and bioavailability of iron such as fortification of bread and taking iron supplements in this age group had been effective; however, these interventions should be continued as well as more especial attention to the treatment and prevention of parasite infestation in this area.
